# Deciphering hematopoietic stem cell heterogeneity: breakthroughs in functional subpopulations and molecular markers

**DOI:** 10.1097/BS9.0000000000000242

**Published:** 2025-06-19

**Authors:** Yangyang Lei, Mei Guo, Huisheng Ai

**Affiliations:** a Senior Department of Hematology, The Fifth Medical Center of PLA General Hospital, Beijing, China; b Chinese PLA General Hospital, Beijing, China

Hematopoietic stem cell transplantation (HSCT) is a primary curative approach for hematological malignancies and genetic diseases, in which hematopoietic stem cells (HSCs) with self-renewal capacity and extremely high pluripotency play a central role.^1^ A large number of studies have demonstrated that HSCs exhibit obvious heterogeneity, which is similar to athletes with different capabilities.^2^ In HSCT, this heterogeneity determines the success of the transplantation, as well as the speed and quality of hematopoietic reconstitution. Therefore, screening out those “champion-class HSCs” with special or super capabilities has always been a difficult problem for decades.^3,4^ Dong et al^5^ employed large-scale and repetitive mouse transplant experiments combined with single-cell transcriptomic sequencing, immunophenotyping, and Bayesian dynamic model to systematically analyze the kinetic patterns of hematopoietic recovery following single-HSC transplantation, identified a “Super”-class HSC clone with exceptional transplantability, and further uncovered CD27 as a key molecular marker. By tracking the hematopoietic reconstitution trajectories of 288 mouse-derived single HSCs over 1 to 4 months post-transplant, they identified three distinct HSC clones: “Super” cluster, “Flash” cluster, and “Trickle” cluster. Remarkably, the “Super” cluster, comprised only 4% of total HSCs, exhibited sustained multilineage hematopoietic capacity through serial transplants, maintaining balanced myeloid/lymphoid lineage outputs even across consecutive generations. The Bayesian dynamic model provided a quantifiable method for dissecting HSC heterogeneity, which help to overcome the limitations of surface marker-based or single-timepoint analyses. A hallmark of “Super”-class HSC clone was their balanced myeloid/lymphoid differentiation potential, critical for post-transplant hematopoietic recovery and immune reconstitution.^6^ Because both granulocytic and lymphocytic lineage recovery are key to establishing effective anti-infection and immune tolerance capabilities after HSCT, while bias lineage reconstitution may lead to complications such as long-term immunodeficiency, graft-versus-host disease (GvHD), and primary or secondary engraftment failure.^7^ Both “Super” cluster and “Flash” cluster showed high multilineage potential in the first generation. However, “Super” cluster sustained balanced output of myeloid/lymphoid lineages in the second and third generations, while the “Flash” cluster clone exhibited biased lineage differentiation. This stability was linked to a unique molecular program that contains self-renewal and differentiation. Unlike Dykstra’s “β”-type reconstitution,^8^ which focused on transplant model in single-generation, “Super”-class HSC clone demonstrated intergenerational consistency in serial transplants. Transcriptome analysis further showed that the key HSC self-renewal regulatory genes such as Socs2, organophosphate biosynthesis-related genes such as Prps1 and Cept1, and phosphatidylinositol 3-kinase (PI3K) negative regulatory genes such as Eng, were enriched in the “Super”-class HSCs. These genes might determine the maintenance of multilineage balance by inhibiting excessive differentiation signals, forming a unique molecular network to coordinate the dynamic equalization between differentiation and proliferation. Single-cell transcriptome analysis revealed the unique signature of the “Super”-class HSC clone. By comparing gene expression across different subclusters in the serial transplants, four differentially expressed gene (DEG) signatures were identified: “Super” (enriched in self-renewal pathways), “Flash” (inflammatory response and leukocyte migration), “Non-Super” (hematopoiesis regulation, myeloid cell differentiation and cell cycle), and “Non-Flash” (nucleic acid metabolism and mitochondrial function). Interestingly, the difference in CD27 expression was the most significant: CD27 expression was significantly lower in the “Super” cluster than in the “Flash” and “Trickle” clusters. Further functional validation experiments showed that CD27⁻ HSCs exhibited significantly superior reconstitution capacity compared to CD27⁺ HSCs in transplants. This was consistent with current research focuses on the manipulation of HSCs, indicating that the quality and efficacy of HSCs, rather than cell number, are key to the success of transplant.^9^ As a membrane surface molecule, low CD27 expression was expected to become a critical indicator for predicting the high “transplantability,” providing a new target for the precise screening of high-potential HSCs.

In summary, Dong et al^5^ analyzed the heterogeneity of HSCs and identified a “Super”-class HSC clone through a series of research methods, which may provide new insights for stem cell modification approaches in HSCT and the development of “precision transplantation strategies.” However, these findings were derived from mouse transplant model, whether “super” class HSCs, including CD27 as a key marker, can be applied to studies of human HSCs (derived from umbilical cord blood, bone marrow, and peripheral blood) still requires further validation and more research work.
